# 

**Published:** 1883-10

**Authors:** 


The Bead Suture; A Modification of the Quilled Suture for Palatoplasty and for Use in Other Situations in which the Suture Line Requires to be Supported for Several Days. By David Prince, M.D., of Jacksonville, Ill.

This suture appears to us as an invention exceedingly interesting to gynecologists.

